# Therapeutic Risk and Benefits of Concomitantly Using Herbal Medicines and Conventional Medicines: From the Perspectives of Evidence Based on Randomized Controlled Trials and Clinical Risk Management

**DOI:** 10.1155/2017/9296404

**Published:** 2017-04-11

**Authors:** Xiu-lai Zhang, Meng Chen, Ling-ling Zhu, Quan Zhou

**Affiliations:** ^1^Division of Medical Administration, The Second Affiliated Hospital, School of Medicine, Zhejiang University, Hangzhou, Zhejiang, China; ^2^Department of Pharmacy, The Second Affiliated Hospital, School of Medicine, Zhejiang University, Hangzhou, Zhejiang, China; ^3^VIP Care Ward, Division of Nursing, The Second Affiliated Hospital, School of Medicine, Zhejiang University, Hangzhou, Zhejiang, China

## Abstract

Despite increased awareness of the potential of herb-drug interactions (HDIs), the lack of rigorous clinical evidence regarding the significance provides a challenge for clinicians and consumers to make rational decisions about the safe combination of herbal and conventional medicines. This review addressed HDIs based on evidence from randomized controlled trials (RCTs). Literature was identified by performing a PubMed search till January 2017. Risk description and clinical risk management were described. Among 74 finally included RCTs, 17 RCTs (22.97%) simply addressed pharmacodynamic HDIs. Fifty-seven RCTs (77.03%) investigated pharmacokinetic HDIs and twenty-eight of them showed potential or actual clinical relevance. The extent of an HDI may be associated with the factors such as pharmacogenomics, dose of active ingredients in herbs, time course of interaction, characteristics of the object drugs (e.g., administration routes and pharmacokinetic profiles), modification of herbal prescription compositions, and coexistence of inducers and inhibitors. Clinical professionals should enhance risk management on HDIs such as increasing awareness of potential changes in therapeutic risk and benefits, inquiring patients about all currently used conventional medicines and herbal medicines and supplements, automatically detecting highly substantial significant HDI by computerized reminder system, selecting the alternatives, adjusting dose, reviewing the appropriateness of physician orders, educating patients to monitor for drug-interaction symptoms, and paying attention to follow-up visit and consultation.

## 1. Introduction

Multimorbidity is the principal cause of complex polypharmacy, which in turn is the prime risk factor for inappropriate prescribing and adverse drug reactions and events [1]. Polypharmacy is not a problem in itself, but there is a risk of drug-drug interactions (DDIs) or herb-drug interactions (HDIs) in the event of poor awareness or a lack of coordination among care providers. Real or potential DDIs and HDIs are one of the key elements in reviewing appropriateness of physician orders, as required by Joint Commission International [2].

At least 16% of US population, 19.2% of Turkish elderly, and 14.1% of Taiwanese residents use prescription drugs and herbal medicines and supplements [3–5]. Despite increased awareness of the potential of HDIs, the lack of rigorous clinical evidence regarding the significance provides a challenge for clinicians and consumers to make rational decisions about the safe combination of herbal and conventional medicines. Potential interactions should be assessed critically for their clinical relevance. For example, coprescribing of low-dose aspirin with herbs is common for patients with cardiocerebrovascular diseases. Pharmacists are usually consulted by patients and clinical professionals for confirming whether combination use of aspirin and* Ginkgo biloba* is appropriate. The addition of* Ginkgo biloba* extract resulted in spontaneous hyphema in a 70-year-old man receiving maintenance therapy of aspirin and probable interaction between aspirin and* Ginkgo biloba* was suspected [6]. PubMed database retrieval till January 2017 identified only one randomized controlled trial of low-dose aspirin-*Ginkgo biloba* interaction. Results of this study showed that there were no adverse bleeding events and potential adverse effects of concomitant use on platelet function in patients with peripheral artery disease or risk factors for cardiovascular disease [7].

The randomized controlled trial (RCT) is considered to provide the most reliable evidence on the effectiveness of interventions because the processes used during the conduct of an RCT minimize the risk of confounding factors influencing the results [8]. There are several reviews on HDIs [9, 10]; however, a review has not been available to address HDIs from the perspective of evidence based on RCTs. Therefore, we here present an updated narrative review on this issue and propose relevant clinical risk management to enhance rational combination use of herbal medicines and conventional medicines.

## 2. Methods

Relevant literature was identified by performing a PubMed search till Jan 2017, using a query “(herb or herbal or traditional Chinese medicine or natural product) and drug interaction” with a filter of “language: English; article type: randomized controlled trials.” Four hundred and four articles were detected. Inclusion criteria included HDIs studies in the field of pharmacokinetics and pharmacodynamics. After reviewing the abstracts, 309 articles were directly excluded because of actually irrelevant topics. Another 21 articles were excluded including food-related (e.g., grapefruit juice, pomegranate juice, and pomelo) studies (*n* = 19) and animal pharmacokinetic studies (*n* = 2). Seventy-four articles were finally included under this search strategy and inclusion/exclusion criteria. The full text of each included article was critically reviewed, and valuable information was summarized by data interpretation.

## 3. Results and Discussion

### 3.1. General Information

Among 74 finally included RCTs, 17 RCTs (22.97%) addressed HDIs simply from the perspective of pharmacodynamics. Eight RCTs revealed either beneficial (*n* = 7) or deleterious (*n* = 1) effects of coadministration of herbal medicines on adverse drug reactions induced by conventional medicines. Four RCTs revealed synergistic efficacy and three RCTs confirmed lower efficacy, whereas the other two RCTs showed no changes in pharmacodynamics when concomitantly using herbal medicines and conventional medicines. It seems that more HDIs studies focusing on pharmacodynamics are necessary to be conducted.

Fifty-seven RCTs (77.03%) investigated HDIs mainly from the perspective of pharmacokinetics. Twenty-nine RCTs (50.88%) directly investigated the pharmacokinetic changes of specific drugs in the presence of comedicated herbal medicines, and fourteen of them showed significant pharmacokinetic HDIs with potential or actual clinical relevance. Utilization of a phenotyping probe or cocktail approach seems to be an efficient in vivo screening tool on drug interaction potential of herbal products as inhibitors or inducers of one or multiple metabolizing enzymes and transporters. Twenty-eight RCTs (49.12%) assessed the influences of herbs on activities of cytochrome P450 (CYP) or P-glycoprotein (P-gp) using phenotyping approach, and fourteen of them showed significant changes in CYP or P-gp activity by the addition of herbal medicines.

Regarding participants enrolled in RCTs of HDIs, fifty-six RCTs (75.68%) were performed in healthy volunteers compared to eighteen RCTs (24.32%) conducted in patients.

**Figure 1:**
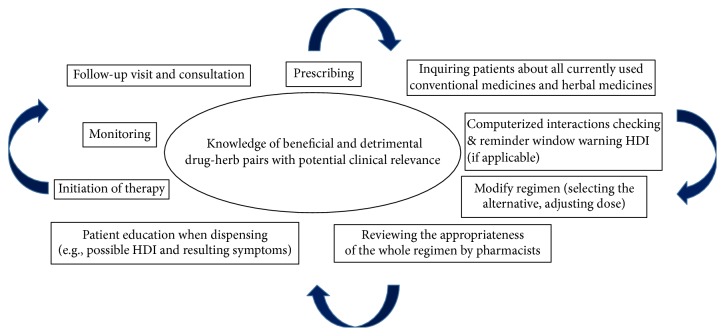
Framework for rational combination use of conventional medicines and herbal medicines.

Table 1 listed pharmacokinetic or pharmacodynamic HDIs with potential or actual clinical relevance and those neutral HDIs lack of potential clinical relevance. Because none of herb-drug pairs was listed as contraindications of conventional medicines involved in this review, general framework for deciding how advice can be developed includes as follows. (1) Do not concomitantly use a herbal medicine if it can significantly reduce plasma concentrations and/or efficacy of an object drug which is expensive (e.g., cyclosporine and rosuvastatin) or it can significantly alter plasma concentrations and/or sensitivity of an object drug which has a narrow therapeutic index (e.g., warfarin). (2) Select an alternative of object drug which is susceptible to an HDI precipitated by a herbal medicine. (3) Caution should be exercised (e.g., dose titration and medication monitoring) if it is unavoidable to use herb-drug pairs exhibiting HDI with potential clinical relevance. Clinical change should be warranted when pharmacodynamic evidence of interaction is sufficiently significant during treatment. The flow chart for rational combination use of conventional medicines and herbal medicines was illustrated in Figure 1.

### 3.2. Significant Pharmacokinetic HDIs with Potential or Actual Clinical Relevance

#### 3.2.1. Herb-Specific Drug Combination

*Baicalin-Rosuvastatin*

*HDI and Risk Description*. Coadministration of baicalin tablets 500 mg three times daily (t.i.d.) for 14 days in healthy participants could significantly decrease the area under the plasma concentration-time curve from zero to infinity (AUC_(0–infinity)_) of rosuvastatin 20 mg by 41.9 ± 7.19% in subjects with organic anion transporting polypeptide 1B1 (OATP1B1)^*∗*^1b/^*∗*^1b (*P* < 0.01), 23.9 ± 8.66% in OATP1B1^*∗*^1b/^*∗*^15 (*P* < 0.01), and 1.76 ± 4.89% in OATP1B1^*∗*^15/^*∗*^15 (*P* > 0.05), respectively. The attenuating effect of baicalin on plasma concentrations and AUC_(0–infinity)_ of rosuvastatin was OATP1B1 haplotype-dependent [11].

*Clinical Risk Management*. To avoid potential reduced efficacy of rosuvastatin, concomitant use of baicalin and rosuvastatin is not suggested in subjects without OATP1B1^*∗*^15 allele. Physicians should consider removing the baicalin from therapeutic regimen when lower efficacy of rosuvastatin is observed in patients concurrently receiving baicalin and rosuvastatin. Given that this RCT only addressed the pharmacokinetic HDI between multidose baicalin and single-dose rosuvastatin in healthy subjects, it is necessary to study pharmacokinetic and pharmacodynamic interaction between baicalin and rosuvastatin in true patients receiving maintenance therapy of rosuvastatin. Also, it is worthy to investigate whether baicalin poses risk of HDIs with other statins. Pharmacokinetic interaction study showed that baicalin had inhibitory effects on the expression and activity of CYP3A in rats [12], and thus potential HDIs may exist when concomitantly using baicalin and CYP3A4-metabolized statins.

*St. John's Wort-Warfarin*

*HDI and Risk Description.* An RCT using population pharmacokinetic-pharmacodynamic modeling approach revealed that coadministration of St. John's wort at recommended daily doses (data not shown) for 7 days significantly increased apparent clearance of S-warfarin but had no direct effect on warfarin pharmacodynamics in healthy subjects [13]. However, another RCT showed that pretreatment with St. John's wort (equivalent to standardized dry extract equivalent to 1 g* Hypericum perforatum* flowering herb top, 0.825 mg hypericin, and 12.5 mg hyperforin) t.i.d. for 14 days could significantly increase apparent clearance of S-warfarin and R-warfarin with a significant subsequent effect on warfarin pharmacodynamics in healthy subjects [14].

*Clinical Risk Management*. To avoid potentially supratherapeutic international normalized ratio (INR) and anticoagulant treatment failure, concomitant use of St. John's wort with warfarin is not advised. Given that the two RCTs only addressed the pharmacokinetic and pharmacodynamic HDI between multidose St. John's wort and single-dose warfarin in healthy subjects [13, 14], studies in patients receiving maintenance warfarin dosing are warranted to assess the clinical significance of this HDI. Currently, RCTs addressing HDIs between new oral anticoagulants and St. John's wort are unavailable and needed to be investigated.

*St. John's Wort-Oxycodone*

*HDI and Risk Description.* St. John's wort administration 300 mg t.i.d. for 15 days could greatly reduce the plasma concentrations of oral oxycodone 15 mg and significantly decrease the oxycodone AUC_(0–infinity)_ by 50% (*P* < 0.001) and self-reported drug effect in healthy participants [15]. The underlying mechanism is that St. John's wort induces the CYP3A-mediated N-demethylation of oxycodone and produces a decrease in the exposure of oral oxycodone.

*Clinical Risk Management*. Due to risk of subtherapeutic concentrations of oxycodone, attention should be drawn to the concomitant use of St. John's wort and oxycodone. Dose titration may be needed to maintain analgesic action of oxycodone during St. John's wort use. Cutting down oxycodone dose is necessary when St. John's wort treatment is discontinued. Given that this RCT addressed the effect of multidose high hyperforin containing product of St. John's wort on the pharmacokinetics and subject drug effect of single-dose oxycodone in healthy subjects, further studies are needed to extend the results to chronic pain patients receiving oxycodone or verify the HDI when low-hyperforin containing St. John's wort extracts are comedicated. Morphine is metabolized primarily by uridine 5′-diphospho-glucuronosyltransferase (UGT) and St. John's wort did not affect UGT activity in the Swiss Webster mouse [16]. Additionally, St. John's wort could potentiate antinociceptive effects of morphine in mice models of neuropathic pain [17]. Thus, morphine might be an alternative of oxycodone when concomitantly using St. John's Wort.

*St. John's Wort-Oral S-Ketamine*

*HDI and Risk Description.* Oral S-ketamine is an adjuvant in the treatment of chronic pain. St. John's wort 300 mg t.i.d. for 14 days decreased AUC_(0–infinity)_ and peak plasma concentration (*C*_max_) of oral S-ketamine 0.3 mg/kg in healthy subjects by 58% and 66%, respectively (*P* < 0.001) [18]. The underlying mechanism for pharmacokinetic changes is probably by inducing the CYP3A-mediated N-demethylation of ketamine in the intestine and liver.

*Clinical Risk Management*. Comedicated St. John's wort may make usual doses of S-ketamine ineffective, and thus caution should be exercised if their combination use is unavoidable. Further studies are needed to evaluate the true clinical significance of the induction when treating patients with chronic pain.

*St. John's Wort-Cyclosporine*

*HDI and Risk Description.* Administration of two 150 mg capsules of St. John's wort with high content of hyperforin (7 mg per capsule) t.i.d. for 14 days could significantly decrease dose-adjusted AUC_(0–12 h)_ for cyclosporine (monoclonal) and steady-state concentrations in renal transplant patients. Moreover, the hyperforin content of St. John's wort extracts could significantly affect the extent of the pharmacokinetic HDI. Coadministration of low-hyperforin (0.1 mg per capsule) St. John's wort extract did not significantly affect cyclosporine pharmacokinetics [19]. The underlying mechanism for pharmacokinetic changes is that St. John's wort is a significant inducer of CYP3A4 and of P-gp, both of which are important in the metabolism and absorption of cyclosporine.

*Clinical Risk Management*. To avoid potential allograft rejection, coadministration of St. John's wort extract and cyclosporine should not be suggested. If their combination use is inevitable, carefully monitor the dose of cyclosporine or select St. John's wort product with low hyperforin content. Furthermore, the interaction with cyclosporine also had cost implications because cyclosporine is very expensive and its dosage had to be increased in the presence of St. John's wort.

*St. John's Wort-Irinotecan*

*HDI and Risk Description.* The elevated plasma concentration of active metabolite SN-38 is responsible for the hematological and gastrointestinal toxicity of irinotecan. An RCT showed that St. John's wort treatment 900 mg daily for 18 days with intravenous irinotecan 350 mg/m^2^ in five cancer patients could decrease the plasma concentrations of the active metabolite SN-38 by 42% (*P* < 0.05) [20]. St. John's wort could reduce the dose-limiting toxicities induced by irinotecan in rats and the possible mechanisms involved the pharmacokinetic interaction with irinotecan and St. John's wort's inhibition of proinflammatory cytokines and intestinal epithelium apoptosis [21, 22].

*Clinical Risk Management*. Given that the appropriate starting dose of irinotecan for patients taking St. John's wort has not been defined, patients receiving irinotecan should refrain from taking St. John's wort to avoid substantially reduced plasma levels of SN-38 and deleterious effect on treatment outcomes. Also, it is interesting to investigate the balance of irinotecan's toxicities and anticancer efficacy in the presence and absence of St. John's wort.

*Echinacea-Warfarin*

*HDI and Risk Description.* An RCT showed that coadministration of* Echinacea* tablets 1275 mg four times daily for 2 weeks could significantly increase the apparent clearance of S-warfarin; however, the magnitude of the mean change was small and the comedication could not significantly alter the pharmacodynamic response to warfarin in healthy subjects [23].

*Clinical Risk Management*. Due to the narrow therapeutic index of warfarin, caution should be exercised when concomitantly using* Echinacea* and warfarin. Given that this RCT only addressed the HDI between multidose* Echinacea* and single-dose warfarin in healthy subjects and that the relatively small sample size for each CYP2C9 or VKORC1 genotype limited interpretation of relationship between genotype and warfarin-*Echinacea* interaction, it is necessary to assess the possibility of pharmacodynamic HDI between* Echinacea* and warfarin in patients taking therapeutic doses of warfarin.

*American Ginseng-Warfarin*

*HDI and Risk Description.* A randomized, double-blind, placebo-controlled trial showed that coadministration of American ginseng 1 g twice daily for 2 weeks could significantly decrease the peak INR, INR AUC, peak plasma warfarin concentration, and warfarin AUC in healthy volunteers [24].

*Clinical Risk Management.* Given that this RCT sample consists of young, healthy volunteers in a research setting rather than patients taking therapeutic doses of warfarin, further study is needed to confirm the results in true patients. When prescribing warfarin, doctors should ask patients about American ginseng use. To avoid potentially supratherapeutic INRs and anticoagulant treatment failure, comedicated American ginseng with warfarin is not suggested. Because coadministration of Korean ginseng extract capsules (equivalent to 1 g* Panax ginseng* root and 17.86 mg ginsenosides) t.i.d. for a week did not affect the pharmacokinetics or pharmacodynamics of either S-warfarin or R-warfarin [14], it is interesting to further compare the difference in warfarin associated HDI risk between American ginseng and* Panax ginseng*.

*Ginkgo biloba Extracts-Simvastatin*

*HDI and Risk Description*. Coadministration of* Ginkgo biloba* extract 120 mg twice daily with simvastatin 40 mg once daily for 2 weeks could significantly decrease simvastatin AUC_(0–infinity)_ and *C*_max_ by 36% and 32%, respectively, but it could not affect the pharmacokinetics of simvastatin acid (active metabolite) and cholesterol-lowering efficacy in healthy volunteers [25].

*Clinical Risk Management.* Given that this RCT only addressed* Ginkgo biloba*-simvastatin interaction in healthy subjects and that* Ginkgo biloba* extract is usually concomitantly administrated with statins to treat diseases in geriatric patients, it is necessary to assess the possibility of pharmacodynamic interaction of* Ginkgo biloba* extract in patients taking simvastatin or in circumstance of higher dosage (i.e.,* Ginkgo biloba* 360 mg/day and simvastatin 80 mg/day). Cutting down simvastatin dose may be necessary to avoid increased risk of myopathy induced by rebounding simvastatin serum concentration when coadministered* Ginkgo biloba* treatment is discontinued. Also, RCTs of HDIs associated with* Ginkgo biloba* extract and other statins are unavailable and are worthy to be conducted.

*Eurycoma longifolia Extract-Propranolol*

*HDI and Risk Description.* Concurrent treatment with propranolol and* Eurycoma longifolia* is possible because hypertensive patients receiving propranolol may experience sexual dysfunction and* Eurycoma longifolia* is a herb commonly consumed for its aphrodisiac properties. An RCT showed that coadministration of a single dose of water-based extract of* Eurycoma longifolia* 200 mg and propranolol 80 mg could significantly decrease the bioavailability of propranolol; that is, AUC_(0–infinity)_ was decreased by 29%, *C*_max_ was decreased by 40%, and time to *C*_max_ (*T*_max_) was prolonged by 86% [26]. The underlying mechanism may be a reduction in absorption rather than an increase in biotransformation. The observed pharmacokinetic changes did not affect the drug's pharmacodynamics in healthy volunteers (e.g., changes in heart rate or blood pressure).

*Clinical Risk Management*. Despite the fact that this RCT only explored the interaction following single-dose regimens of propranolol and* Eurycoma longifolia* in healthy volunteers, caution may still be warranted with coadministering multiple doses of the herb and propranolol to patients.

*Yin Zhi Huang-Omeprazole*

*HDI and Risk Description.* Yin Zhi Huang, a decoction of Yin Chin (*Artemisia capillaris*) and three other herbs, is widely used in Asia to prevent and treat jaundice. Pretreatment of Yin Zhi Huang 10 mL t.i.d. for 14 days induced both CYP3A4-catalyzed sulfoxidation and CYP2C19-dependent hydroxylation of omeprazole, leading to marked decreases in plasma omeprazole concentrations in healthy volunteers [27].

*Clinical Risk Management*. Yin Zhi Huang, acting as an inducer of both CYP3A4 and CYP2C19, may lead to therapeutic failure or insufficient curative effect toward omeprazole and their combination use should be avoided. Pantoprazole and rabeprazole may be alternatives to omeprazole [28]. Also, it is necessary to determine whether pharmacokinetics and pharmacodynamics of other CYP2C19 substrates are interfered by combination use of Yin Zhi Huang.

*Dehusked Garcinia kola Seeds-Quinine*

*HDI and Risk Description.* Coadministration of quinine and* Garcinia kola* (12.5 g once daily or 12.5 g twice daily) for 7 days in healthy Nigerian volunteers resulted in significant pharmacokinetic changes of quinine (i.e., *C*_max_ decreased by 19% and 26%, resp.; *T*_max_ was prolonged by 27% and 48%, resp.), without altering the half-life and apparent clearance of quinine. Meanwhile, the incidences of adverse reactions to quinine were greatly reduced when* Garcinia kola* was coadministered with quinine [29]. The underlying mechanism for this HDI may be absorption interference rather than CYP3A-mediated metabolic interaction.

*Clinical Risk Management*. To avoid a potential to alter the efficacy of oral quinine therapy, caution may need to be exercised with the ingestion of* Garcinia kola* together with oral quinine. Also, it is interesting to design a regimen exhibiting that coadministered* Garcinia kola* reduces adverse effects while not lowering antimalaria efficacy of quinine. Because quinine is a typical CYP3A4 substrate, this RCT indicates that CYP3A activity in humans is not significantly affected in the presence of* Garcinia kola*.

*Berberine-Cyclosporine*

*HDI and Risk Description.* A randomized and controlled clinical trial showed that berberine could markedly elevate the blood concentration of cyclosporine in renal transplant recipients. After 3 months of combination therapy of berberine (0.2 g t.i.d) and cyclosporine, 52 berberine-treated renal transplant recipients experienced higher final blood concentrations and ratios of concentration/dose of cyclosporine compared to 52 berberine-free patients (*P* < 0.05), with the incremental percentage being 29.3% and 27.8%, respectively. Additionally, a pharmacokinetic study showed that coadministration of berberine 0.2 g t.i.d. and cyclosporine 3 mg/kg twice daily for 12 days in six renal transplant recipients increased the mean AUC of cyclosporine by 34.5% and the steady-state minimum blood concentration by 88.3% (*P* < 0.05) while significantly reducing apparent clearance by 40.4% and the peak-to-through fluctuation index by 50%. There were no significant alternations in routine blood chemistry and hepatic and renal functions before and after coadministration of berberine [30]. The underlying mechanism for berberine-cyclosporine interaction may be attributed to the inhibitory effects of berberine on intestinal P-gp and CYP3A in the liver and small intestine.

*Clinical Risk Management*. This HDI may be possible to allow a reduction of the cyclosporine dosage and thus it is interesting to further investigate the potential economic benefit brought by the addition of berberine to cyclosporine treatment in renal transplant recipients (e.g., a reduction in cyclosporine dosage and less frequency of adverse drug reactions).

#### 3.2.2. Effects of Herbal Medicines on CYP and/or Transporters Based on Phenotyping Study

*Effect of St. John's Wort on CYP3A*

*HDI and Risk Description.* Pretreatment of St. John's wort preparations for 14 days could decrease the oral AUC of CYP3A probe midazolam and induce CYP3A activity, although the extent of the inducible effect correlated significantly with increasing hyperforin content. Elimination half-life was not significantly changed or met the bioequivalence limits even in the group with the largest midazolam AUC and *C*_max_ reduction, indicating that St. John's wort's interaction probably affected mainly intestinal CYP3A rather than hepatic CYP3A [31]. Phenotyping cocktail approach in healthy volunteers also revealed strong induction of CYP3A by St. John's wort [32, 33].

*Clinical Risk Management*. It is necessary to avoid combination use of St. John's wort and CYP3A substrates. If applicable, those preparations with low hyperforin content could be used due to reduced risk for relevant CYP3A induction and HDIs. Moreover, the effect of St. John's wort could be of limited clinical relevance for CYP3A substrates with a broader therapeutic range or limited intestinal first-pass metabolism.

*Effect of St. John's Wort on P-gp*

*HDI and Risk Description.* Comedicated St. John's wort products varying in dose and formulation for two weeks could exert different effects on pharmacokinetics of P-gp probe digoxin. High-dose hyperforin-rich extract exhibited more prominent reduction in AUC_(0–24)_, steady-state concentrations of digoxin, whereas* Hypericum* powder without hyperforin, tea, juice, oil extract, hyperforin-free extract and hyperforin-containing* Hypericum* powder at low daily doses had no significant interaction with digoxin [34, 35]. St. John's wort 600 mg t.i.d. for 16 days increased P-gp expression 4.2-fold and enhanced the drug efflux function of P-gp in peripheral blood mononuclear cells of healthy volunteers [36].

*Clinical Risk Management*. It is necessary to avoid combination use of St. John's wort and P-gp substrates. When coadministration of P-gp substrates and St. John's wort is unavoidable, patients should be informed about the clinically significant HDI risk and possible resulting symptoms.

*Effect of St. John's Wort on CYP2C19*

*HDI and Risk Description*. Treatment of St. John's wort tablet 300 mg t.i.d. for 14 days significantly increased CYP2C19 activity in CYP2C19 wild-genotype subjects, with mean urinary 4′-hydroxymephenytoin excretion (an index for CYP2C19 activity) increased by 151.5% (*P* < 0.05), whereas no significant alteration was observed for CYP2C19 poor metabolizers [37]. St. John's wort tablet 300 mg t.i.d. for 14 days induced both CYP3A4-catalyzed sulfoxidation and CYP2C19-dependent hydroxylation of omeprazole and greatly decreased the plasma concentrations of omeprazole [38].

*Clinical Risk Management*. Physicians prescribing omeprazole with St John's wort should be aware that treatment may fail as a result of lower levels of omeprazole. Pantoprazole and rabeprazole have lower potential for CYP2C19-mediated interactions and may be alternatives to omeprazole [28]. Caution may need to be exercised when St. John's wort is added to or withdrawn from an existing drug regimen containing CYP2C19 substrates.

*Effect of Ginkgo biloba Extracts on P-gp*

*HDI and Risk Description*.* Ginkgo biloba* extracts ingestion 360 mg once daily for 14 days, rather than a single oral dose, could significantly affect the pharmacokinetics of P-gp probe talinolol in humans; that is, *C*_max_ and AUC_(0–infinity)_ increased by 36%, and 22%, respectively [39].

*Clinical Risk Management.* Caution may need to be exercised when* Ginkgo biloba* extract is added to or withdrawn from an existing drug regimen containing P-gp substrates. Given that this RCT is a preliminary work to discover the potential influence of* Ginkgo biloba* extracts use on the pharmacokinetics of a single oral dose of talinolol in a limited number of male volunteers, it is necessary to perform further RCT investigation of long-term use of* Ginkgo biloba* extracts on the pharmacokinetics of repeated oral administration of P-gp substrates in a larger study population.

*Effect of Rhodiola rosea on CYP2C9*

*HDI and Risk Description*. Phenotyping cocktail approach indicated that pretreatment with* Rhodiola rosea* extract 290 mg daily for 14 days could reduce CYP2C9 activity by 21% without significant effects on CYP1A2, CYP2C19, CYP2D6, and CYP3A4 [40].

*Clinical Risk Management*. It may be clinically relevant during concomitant use of* Rhodiola rosea* extract and CYP2C9 substrates with a narrow therapeutic index (e.g., phenytoin and warfarin). A full pharmacokinetic study with these substrates should be conducted to confirm and extend the results from Thu et al.'s study [40].

*Effect of Goldenseal on CYP3A and CYP2D6*

*HDI and Risk Description*. Coadministration of goldenseal (1323 mg, t.i.d., standardized to contain 24.1 mg isoquinoline alkaloids per capsule) for 14 days could significantly alter pharmacokinetic parameters of CYP3A probe midazolam (mean AUC_(0–infinity)_ increased by 62.5%, apparent clearance decreased by 35.7%, and *C*_max_ increased by 40.7%), indicating its inhibitory effect on CYP3A activity. This RCT used rifampin and clarithromycin as positive controls for CYP3A induction and inhibition, respectively; therefore the clinical significance of supplement-mediated interactions could be well gauged [41]. Phenotyping cocktail approach revealed significant inhibition of CYP2D6 and CYP3A (approximately 40%) following goldenseal administration 900 mg t.i.d. for 28 days [42].

*Clinical Risk Management*. Potentially serious adverse interactions may result from combination use of goldenseal supplements and substrates of CYP2D6 or CYP3A. However, a comparative study showed that treatment with goldenseal root (1140 mg twice daily) for 14 days had no influence on the pharmacokinetics of CYP3A4 substrate indinavir in healthy volunteers. Indinavir is not a good substrate for assessing gut wall extraction due to its relatively high oral bioavailability [43]; therefore it indicates that oral bioavailability of drugs that undergo extensive first-pass metabolism by CYP3A in the gut wall is more susceptible to goldenseal inhibition.

*Effect of Genistein on CYP3A and P-gp*

*HDI and Risk Description*. A placebo-controlled randomized study showed that genistein administration 1000 mg once daily for 14 days could decrease the systemic exposure of midazolam and talinolol in eighteen healthy volunteers, suggesting statistically significant induction of CYP3A and P-gp activity [44].

*Clinical Risk Management*. Caution may need to be exercised when comedicating genistein with CYP3A or P-gp substrates, especially those with a narrow therapeutic index. Also, it is necessary to perform further investigation of long-term use of genistein on the pharmacokinetics of repeated oral administration of CYP3A and P-gp substrates in a larger study population.

*Effect of Berberine on CYP2D6, CYP2C9, and CYP3A4*

*HDI and Risk Description*. Phenotyping cocktail approach in 17 healthy male volunteers revealed that administration of berberine 300 mg t.i.d. for two weeks could significantly decrease the activities of CYP2D6, CYP2C9, and CYP3A4 rather than CYP2C19 and CYP1A2 [45].

*Clinical Risk Management.* This RCT provides partial evidence for explaining berberine-cyclosporine (CYP3A4 substrate) interaction observed in Wu et al.'s RCT [30]. HDIs need to be considered when berberine or berberine-containing products are concurrently administered with substrates of CYP2D6, CYP2C9, or CYP3A4. However, the usual dose of berberine is ranging from 100 mg to 300 mg t.i.d. and it is unclear whether berberine 100 mg t.i.d. has a significant effect on activities of CYP2D6, CYP2C9, or CYP3A4. Further studies with larger sample size and genotypes for relevant CYPs will be helpful to confirm and expand the results of Guo et al.'s study [45].

### 3.3. Pharmacodynamic HDIs with Potential or Actual Clinical Relevance

#### 3.3.1. Effects of Herbal Medicines on Adverse Drug Reactions Associated with Conventional Medicines

*Free and Easy Wanderer Plus-Carbamazepine*. Free and Easy Wanderer Plus (FEWP) is a well-known traditional Chinese medicine for treatment of various mood disorders. A double-blind placebo-controlled randomized study showed that adjunctive FEWP therapy (36 g/day) with carbamazepine could improve the tolerability of carbamazepine in the 26-week treatment of mood disorders (i.e., a significantly lower overall discontinuation rate, fewer side effects, and lower serum levels of carbamazepine) [46].

*Shakuyaku-kanzo-to-Antipsychotic Agents*. Traditional Japanese herbal medicine shakuyaku-kanzo-to 7.5 g daily for 2 weeks was useful in decreasing extrapyramidal symptom in ten patients undergoing treatment with antipsychotic agents [47]. Further investigations with a larger number of patients and longer intervention and follow-up periods are needed to expand on the findings of this preliminary research.

*Saffron Aqueous Extract-Olanzapine or Fluoxetine*. Saffron aqueous extract 30 mg daily for 12 weeks could prevent metabolic syndrome and increases in blood glucose in schizophrenia patients on olanzapine treatment [48]. Saffron 15 mg twice daily for 4 weeks could significantly improve fluoxetine-related sexual dysfunction among both female and male patients with major depressive disorder who were stabilized on fluoxetine [49, 50].

*Ginkgo biloba Extract-Haloperidol*.* Ginkgo biloba* extract 360 mg daily for 12 weeks could significantly enhance the efficiency of haloperidol 0.25 mg/kg/day in patients with schizophrenia, especially on their positive symptoms. The underlying mechanism may be associated with* Ginkgo biloba* extract's antioxidant effect that is involved in the therapeutic mechanism in patients with chronic refractory schizophrenia [51].

*Cranberry-Warfarin*

*HDI and Risk Description*. Treatment with cranberry juice concentrate capsules 1000 mg t.i.d. (equivalent to 57 g of fruit per day) for 2 weeks significantly increased the sensitivity of healthy subjects to warfarin without influencing the pharmacokinetics of warfarin enantiomers [52].

*Clinical Risk Management*. Combination use of warfarin and cranberry may require careful monitoring. Practitioners should consider cranberry usage as a potential contributor in the evaluation of supratherapeutic INR values in patients on warfarin.

*Ginkgo biloba Extract-Iodine-131 Therapy*.* Ginkgo biloba* extract 120 mg daily for 1 month could protect from possible oxidative and genotoxic damage associated with iodine-131 therapy in ten patients with thyroid cancer, without any adverse modification of the clinical outcome [53]. Further studies with larger cohorts of patients or higher doses of* Ginkgo biloba* extract are needed to confirm the beneficial effect in patients requiring iodine-131 therapy, particularly for those in whom repeated treatments and high activities of iodine-131 are needed.

#### 3.3.2. Effects of Herbal Medicines on Efficacy of Conventional Medicines

*Radix/Rhizoma Notoginseng Extract-Aspirin*. Low dose of aspirin (50 mg/day) combined with Radix/rhizoma notoginseng extract (sanchitongtshu capsule) 200 mg t.i.d. had a synergistic action in the treatment of patients with light and moderate ischemic stroke in acute and subacute stages (i.e., ameliorated neurological deficit and activities of daily living and equal frequency of adverse reaction) [54]. Further RCT is necessary to investigate the HDI between Radix/rhizoma notoginseng extract and aspirin (75–150 mg/day). Also, it is interesting to conduct a comparative study of aspirin (50 mg/day) in combination with Radix/rhizoma notoginseng extract versus aspirin (75–150 mg/day) comedicated with placebo.

*Bergamot Polyphenolic Fraction-Rosuvastatin*. Oral bergamot polyphenolic fraction 1000 mg daily for 30 days could enhance rosuvastatin-induced effect on low-density lipoprotein cholesterol (LDL-C), lipoxygenase-1 expression, and protein kinase B phosphorylation in patients with hyperlipidemia [55]. Clinically, addition of bergamot polyphenolic fraction to rosuvastatin may allow a reduction in daily rosuvastatin doses for achieving the target levels of cholesterol.

*Total Ginsenosides-Ulinastatin*. Shenmai injection is mainly made of Red Ginseng and Radix Ophiopogonis and widely used for treating coronary heart disease, organ protection, and adjunct therapy to tumor chemotherapy. Total ginsenosides are the major components of this herb injection. Coadministration of Shenmai injection 100 ml twice daily for 7 days could effectively synergize with intravenous ulinastatin 100,000 units t.i.d. against septic acute lung injury and acute respiratory distress syndrome [56].

*Fuzheng Yiliu Decoction-Chemotherapy*. Fuzheng Yiliu decoction orally administered 3 days before chemotherapy and lasting to the end of 2 cycles of chemotherapy could enhance therapeutic effects of chemotherapy on malignant gastrointestinal tumor and reduce the toxic and side effects on bone marrow and digestive tract [57].

*St. John's Wort-Oral Contraceptive*

*HDI and Risk Description*. An RCT indicated no evidence of ovulation among healthy females during combination therapy of St. John's wort extract 500 mg to 900 mg daily and low-dose oral contraceptive (0.02 mg ethinyl estradiol and 0.15 mg desogestrel), but coadministration increased intracyclic bleeding episodes, adversely affected compliance to oral contraceptives, and decreased serum 3-ketodesogestrel concentrations, which may potentially enhance the risk of unintended pregnancies [58].

*Clinical Risk Management*. St. John's wort extracts should be used with caution in women taking oral contraceptive. This RCT only studied the interaction of St. John's wort extract with only one preparation of oral contraceptive and therefore further interaction studies with other oral contraceptives consisting of different combinations of hormones should be conducted. Meanwhile, a large clinical trial with a longer follow-up period is required to overcome the limitation of this RCT (i.e., relatively small sample size).

*St. John*'*s Wort-Atorvastatin or Simvastatin*

*HDI and Risk Description*. Coadministration of St. John's wort product (containing 300 mg* Hypericum perforatum*) twice daily for 4 weeks significantly increased the serum level of LDL-C and total cholesterol compared with control (a commercially available multivitamin tablet) in hypercholesterolemic patients receiving atorvastatin or simvastatin for at least 3 months [59, 60]. Results of these two RCTs may be of clinical importance because atorvastatin efficacy reduced by 30% [59], and about half of the effect of simvastatin on LDL-C was lost in patients [60]. The most likely explanation for pharmacodynamic changes is pharmacokinetic interactions between St. John's wort (a CYP3A4 and P-gp inducer) and atorvastatin or simvastatin (two substrates for CYP3A4 and P-gp).

*Clinical Risk Management*. The general recommendation to patients ought to be to avoid the combination of St. John's wort product and two statins. Patients should be checked for their lipids more carefully or there is a need for increasing the dose of atorvastatin and simvastatin if combined therapy with St. John's wort is necessary.

### 3.4. Neutral HDIs Lack of Potential Clinical Relevance

#### 3.4.1. Evidence Based on Phenotyping Cocktail Approach

Liu Wei Di Huang Wan, a well-known traditional Chinese patent medicine (containing Radix Rehmanniae, pulp of* Cornus*, yam,* Poria cocos*,* Alisma orientale*, and Cortex Moutan), is widely used for the treatment of various diseases in China. Phenotyping cocktail approach revealed that coadministration of Liu Wei Di Huang Wan (12 pills, 0.2 g/pill, twice daily) for 14 days had no effect on the activities of CYP2C19, CYP2D6, and CYP3A4 in healthy volunteers. Liu Wei Di Huang Wan could be unlikely to cause pharmacokinetic interaction when comedicated with conventional medicines predominantly metabolized by these enzymes [61].

Cocktail approach indicated no significant effect on CYP1A2, CYP2D6, CYP2E1, and CYP3A4 activity in healthy volunteers by pretreatment of* Citrus aurantium* (350 mg, twice daily, standardized to 4% synephrine),* Echinacea purpurea* (800 mg, twice daily), milk thistle (175 mg, twice daily, standardized to 80% silymarin), saw palmetto extracts (160 mg, twice daily, standardized to 85% to 95% fatty acids and sterols),* Ginkgo biloba* (60 mg, four times daily, standardized to 24% flavone glycosides and 6% terpene lactones), or* Panax ginseng* pretreatment (500 mg, t.i.d., standardized to 5% ginsenosides) for 28 days [32, 33, 62].

A commercially available curcuminoid/piperine extract (4 g curcuminoids plus 24 mg piperine) orally four times over 2 days did not significantly affect activities of CYP3A, CYP2C9, and the paracetamol conjugation enzymes (dual UGT and sulfotransferase probe) in human volunteers. However, this RCT only treated subjects for 2 days; whereas curcumin would be used clinically for a longer period in human patients, future study should evaluate the effects of more chronic dosing on enzyme function [63]. A low-hyperforin St. John's wort extract product 240 mg (containing 3.5 mg hyperforin) for 10 days did not significantly affect kinetics of investigated probe drugs (alprazolam, caffeine, tolbutamide, and digoxin) in healthy volunteers, indicating negligible modulatory effect on CYP3A4, CYP1A2, CYP2C9, and P-gp [64].

#### 3.4.2. Evidence Based on Single Probe for CYP Phenotyping

Woohwangcheongsimwon suspension, a commonly used herbal medicine in Korea and other East Asian countries for treating hypertension, arteriosclerosis, coma, and stroke, has a negligible effect on the pharmacokinetics of bupropion and 4-hydroxybupropion following a single dose of 150 mg bupropion in healthy volunteers, indicating little inhibitory effect on CYP2B6 activity in vivo. Dosage adjustment of bupropion is unnecessary in patients temporarily concomitantly receiving the highest recommended daily dose of woohwangcheongsimwon suspension [65]. A standardized* Ginkgo biloba* leaf preparation (3 doses of 120 mg) did not alter clearance of flurbiprofen in healthy volunteers, indicating no modulatory effects on CYP2C9 activity and no risk of interactions with drugs extensively metabolized by CYP2C9. However, this RCT involved only very short-term exposure to* Ginkgo biloba* and therefore would not have demonstrated CYP modulatory effect of this herbal medicine [66]. A standardized milk thistle (900 mg) or black cohosh (80 mg) supplement for 14 days did not affect oral midazolam pharmacokinetics, indicating no clinically relevant effect on CYP3A activity in vivo. This RCT utilized rifampin and clarithromycin as positive controls for CYP3A induction and inhibition, respectively; therefore the clinical relevance of supplement-mediated interactions could be well gauged [67]. Administration of silymarin (two doses of 280 mg) or turmeric extract (one dose of 480 mg) did not significantly change the pharmacokinetics of nifedipine (CYP3A4 probe drug) in healthy volunteers. These two RCTs utilized one or two doses of precipitant herbal medicines and therefore could not reflect the true clinical circumstance of chronic dosing. Further RCTs are necessary to address whether the two herbs are not potent CYP3A4 inhibitors in vivo following longer-term pretreatment [68, 69].

Grape seed extract is supposed to have beneficial effects on the cardiovascular system and exert chemopreventive effects in several types of cancer including breast cancer. Consumption of grape seed extract 100 mg t.i.d. for three days did not significantly affect the urinary dextromethorphan to dextrorphan metabolic ratio (a CYP2D6 phenotyping index) in healthy volunteers. This RCT excluded poor metabolizers from the pharmacokinetic analysis by prescreening of CYP2D6 genotyping. With sufficient statistical power, the study indicated that it would be unlikely to have significant interactions with drugs extensively metabolized by CYP2D6 (e.g., metoprolol and tamoxifen) [70].

#### 3.4.3. Evidence Based on P-gp Phenotyping

Administration of Radix Astragali extract granules 4 g (standardized to astragaloside IV 6 mg) twice daily did not have a statistically significant impact on systematic exposure to fexofenadine in healthy volunteers, suggesting that Radix Astragali extract is not a potent modulator of P-gp in vivo [71]. Concomitant therapy of* Crataegus* special extract 450 mg twice daily and digoxin 0.25 mg for three weeks did not significantly change the pharmacokinetics of digoxin in healthy volunteers [72]. Two RCTs compared supplement effects to those of rifampin (a P-gp inducer) and clarithromycin (a P-gp inhibitor) as a means of gauging the clinical relevance of supplement-mediated interactions [73, 74]. Consumption of standardized goldenseal (3210 mg daily), kava-kava (1227 mg daily), milk thistle (900 mg daily), or black cohosh (40 mg daily) supplement for 14 days did not affect digoxin pharmacokinetics, suggesting no potent modulatory effect of two supplements on P-gp in healthy volunteers. These results may not extend to regimens utilizing higher dosages or longer supplementation periods.

#### 3.4.4. Herb-Specific Drug Combination

*St. John's Wort*. St. John's wort 325 mg t.i.d. for 14 days did not significantly affect the pharmacokinetics and pharmacodynamics of repaglinide in healthy volunteers, suggesting that St. John's wort can be administered with repaglinide without any clinical consequences [75]. However, larger sample size clinical trials in patients with type 2 diabetes mellitus are necessary to be conducted to confirm this conclusion. St. John's wort extract 600 mg once daily for 14 days did not remarkably alter plasma concentrations of boceprevir and its metabolite in healthy volunteers, suggesting that St. John's wort and boceprevir can be safely coadministered. However, the study has one limitation; that is, it was conducted in healthy volunteers who may differ from hepatitis C virus-infected individuals with hepatic impairment [76].

*Ginkgo biloba Extracts. *Single-dose pharmacokinetics of cilostazol, voriconazole, or raltegravir in healthy volunteers were not significantly altered following pretreatment of* Ginkgo biloba* extracts 80 mg twice daily for 7 days, 120 mg twice daily for 12 days, or 120 mg twice daily for 15 days, respectively [77–79]. A single dose of* Ginkgo biloba* extract 80 mg, 120 mg, or 240 mg did not significantly alter the pharmacokinetics and antiplatelet activity of ticlopidine, cilostazol, and clopidogrel in healthy volunteers [80, 81].* Ginkgo biloba* extracts (equivalent to 4 g of* Ginkgo biloba* leaf, 19.2 mg of* Ginkgo* flavonglycosides, and 4.8 mg of ginkgolides and bilobalide) t.i.d. for 7 days did not significantly affect clotting status and the pharmacokinetics or pharmacodynamics of warfarin in healthy subjects [82]. Although these six RCTs concluded neutral HDI results in healthy volunteers, large trials in patients with a longer time period of herbal coadministration would be necessary to confirm the results.

*Garlic Extract*. Aged garlic extract 5 ml (containing extracted solids 305 g/L and S-allyl cysteine 1.47 g/L) twice daily for 12 weeks posed no serious hemorrhagic risk for closely monitored patients on warfarin therapy [83]. Garlic capsules 10 mg twice daily over 4 days did not significantly alter the single-dose pharmacokinetics of ritonavir in healthy volunteers. However, this RCT only investigated pharmacokinetic changes of single-dose ritonavir after pretreatment of a short duration of garlic therapy [84]. A non-RCT study showed that 3 weeks of garlic had a significant effect on saquinavir pharmacokinetics (e.g., the mean saquinavir AUC during the 8-hour dosing interval decreased by 51%) [85]. The possibility of an interaction between longer-duration garlic and ritonavir at steady-state conditions still needs to be evaluated because of the complex effects of ritonavir and garlic on metabolizing enzyme.

*Miscellaneous*. Coadministration of aqueous extracts of* Panax ginseng* (0.5 g t.i.d.) and warfarin for 2 weeks did not affect warfarin pharmacodynamics in ischemic stroke patients [86]. Ginger extracts (equivalent to 1.2 g of ginger rhizome powder) t.i.d. for 7 days did not significantly affect clotting status, the pharmacokinetics or pharmacodynamics of warfarin, and CYP2C9 activity in healthy volunteers [82]. Milk thistle extract 450 mg t.i.d. for 28 days did not significantly affect indinavir levels in healthy participants [87]. Lavender oil preparation 160 mg daily for 21 days did not affect the pharmacokinetics and pharmacodynamics of a combination oral contraceptive containing ethinyl estradiol 0.03 mg and levonorgestrel 0.15 mg in healthy fertile adult females [88]. The rate and extent of bioavailability of ofloxacin were not altered after single oral coadministration of ofloxacin and each herbal medicine (Sho-saiko-to, Rikkunshito, and Saireito) in healthy volunteers [89]. Administration of four traditional Chinese medicine preparations (Tong Xin Luo, Nao Xin Tong, Guan Mai Ning, or Yin Xing Ye) at recommended doses of package insert for 7 days did not alter the AUC of simvastatin and simvastatin acid following a single dose of 20 mg simvastatin in healthy volunteers [90]. Paeoniae Radix extract 1.2 g once daily for 7 days did not significantly affect valproic acid pharmacokinetics in healthy volunteers [91].

### 3.5. Factors Affecting the Extent of an HDI

#### 3.5.1. Pharmacogenomics

Pharmacogenomics may involve the pharmacokinetic mechanisms to affect the extent of HDIs among populations. For example, Yin Zhi Huang-omeprazole interaction was CYP2C19 genotype-dependent. The decreases in the AUC_(0–infinity)_ ratio of omeprazole to 5-hydroxyomprazole (an index of CYP2C19 activity) following herb pretreatment in CYP2C19^*∗*^1/^*∗*^1 and CYP2C19^*∗*^1/^*∗*^2 or ^*∗*^3 are 1.5 and 1.7 times greater than that of CYP2C19^*∗*^2/^*∗*^2, respectively, indicating a gene-dosage-inductive effect of Yin Zhi Huang on CYP2C19 activity [27]. The inductive effect of St. John's wort on CYP2C19 activity was only observed in CYP2C19 wild-genotype subjects rather than CYP2C19 poor metabolizers [37]. The inhibitory effect of* Rhodiola rosea* on CYP2C9 was more pronounced in CYP2C9 extensive metabolizers than in CYP2C9 intermediate and poor metabolizers [40]. Phenotype of poor CYP2C19 metabolizers occurs in 2%–5% of Caucasian population and in up to 11%–23% of Oriental population [92]. Caucasians possess higher frequencies of CYP2C9^*∗*^3 than Asians (6–10% versus 2–5%), while 8–20% of Caucasians appear to have the CYP2C9^*∗*^2 allele compared to East Asians who rarely carry CYP2C9^*∗*^2 allele [93]. Clinicians should know that the extent and outcome of an HDI due to enzyme induction or enzyme inhibition among extensive metabolizers may differ from that among poor metabolizers.

Baicalin-rosuvastatin interaction was OATP1B1 haplotype-dependent [11]. OATP1B1^*∗*^1B haplotype frequencies in Chinese and European Americans are 59.9% and 30%, respectively; and OATP1B1^*∗*^15 haplotype frequencies in Chinese and Caucasians are 14% and 2.4%, respectively [94, 95]. Our laboratory observed that HDI between Radix Astragali extract and fexofenadine was dependent on ATP binding cassette subfamily B member 1 (ABCB1, the gene encoding for P-gp) C3435T genotype; that is, Radix Astragali extract pretreatment lengthened elimination half-life of fexofenadine in ABCB1 3435CC carriers but not in ABCB1 3435TT and 3435CT carriers. Due to a considerably higher frequency of the ABCB1 3435CC genotype in Africans (83%), Chinese (25%), and Caucasians (26%), cautions may be necessary for those ABCB1 3435CC carriers receiving combined use of Radix Astragali extract formulation and P-gp substrate [71].

#### 3.5.2. Dose of Active Ingredients in Herbs

The extent of induction of CYP3A varies among St. John's wort products with different hyperforin content and depends on hyperforin dose. The extent of midazolam AUC decrease correlated significantly with increasing hyperforin dose [31]. Regarding quinine monotherapy and concurrent therapy with* Garcinia kola*, AUC_(0–infinity)_ of quinine was bioequivalent at the 12.5 g/day dose and not bioequivalent at the 12.5 g twice daily dose of* Garcinia kola*. This suggests that a dose-dependent interaction may have occurred between* Garcinia kola* and quinine [29]. Hyperforin content determined the magnitude of the St. John's wort-cyclosporine drug interaction. Comedication with a typical high-hyperforin extract resulted in a significant 52% decrease in cyclosporine AUC_(0–12 h)_, whereas comedication with a low-hyperforin product caused no significant reduction in the mean cyclosporine AUC_(0–12 h)_ [19]. High-dose hyperforin-rich St. John's wort exhibited more prominent reduction in AUC_(0–24 h)_, peak, and trough concentrations of digoxin [35]. Studies would likely need to be repeated with newer formulations of herbal medicines (such as nanoparticles and liposome encapsulated forms) to determine whether the higher active ingredient bioavailability also enhances the risk for drug interactions.

#### 3.5.3. Time Course of Interaction

HDIs may be time-dependent. For example, a single oral dose of* Ginkgo biloba* extract (120 mg) did not affect the pharmacokinetics of P-gp probe talinolol, whereas repeated ingestion of* Ginkgo biloba* extract (360 mg/day) for 14 days increased the talinolol *C*_max_ by 36% and AUC_(0–infinity)_ by 22%, respectively [39]. During the initial 10 hours of the first day of St. John's wort administration, the voriconazole AUC was increased by 22% compared with control. After 15 days of St. John's wort intake, AUC_(0–infinity)_ was reduced by 59% and oral voriconazole apparent clearance was increased 1.44-fold compared with control [96]. A single dose of St. John's wort significantly increased *C*_max_ of fexofenadine by 45% and decreased the oral clearance by 20%; however, long-term St. John's wort (300 mg t.i.d.) for 2 weeks caused a significant 35% decrease in *C*_max_ and a significant 47% increase in fexofenadine oral clearance. Long-term treatment with St. John's wort reversed the changes in fexofenadine pharmacokinetics following a single-dose administration [97]. The interaction of St. John's wort extract with digoxin pharmacokinetics was time-dependent and the effect became increasingly pronounced until the tenth day of coadministration [34].

Pharmacokinetic modelling of the interaction between St. John's wort and cyclosporine showed that elimination half-life of the detoxicating proteins (e.g., CYP3A4 and P-gp) was 4.4 days, suggesting that a period of 2-3 weeks may be required for normalization of the level of the detoxicating proteins and the dose of cyclosporine should be carefully monitored and modified as necessary for at least 2 weeks after the cessation of St. John's wort intake [98].

#### 3.5.4. Characteristics of the Object Drug

The object drug is defined as the medication whose pharmacokinetics and/or pharmacodynamics may be modified by the drug interaction process. Clinicians should be aware of the fact that the effect of precipitant herb may not be extrapolated from data on agents belonging to the same therapeutic class. For example, St. John's wort 300 mg t.i.d. for 14 days could decrease plasma concentrations of simvastatin but not pravastatin [99]. The underlying mechanism is that St. John's wort strongly induces CYP3A4 which extensively metabolizes simvastatin in the intestinal wall and liver, whereas pravastatin is a non-CYP3A4-metabolized statin with the minimal risk of interaction with CYP3A4 modifiers.

The extent of an HDI may be determined by administration route of the object drug. For example, long-term St. John's wort administration caused a nonsignificant 20% decrease in AUC when midazolam was given intravenously compared to a significant > 50% decrease in AUC when midazolam was given orally [100]. The underlying mechanism is that St. John's wort's selectively induced CYP3A activity in the intestinal wall rather than in the liver and thus it primarily increased the first-pass elimination of midazolam with a lesser and nonsignificant effect on the systemic clearance.

#### 3.5.5. Modification of Herbal Prescription Compositions

Sho-saiko-to, Saiboku-to, and Saireito are Japanese herbal medicines consisting of similar herbal compositions. Saiboku-to and Saireito contain additional herbal extracts in addition to the seven constituents of Sho-saiko-to. Coadministration of prednisone and each herb product 2.5 or 3.0 g t.i.d. for three days resulted in different extents of HDIs; that is, active metabolite prednisolone's AUC significantly decreased from 0.94 to 0.78 mg·h·L^−1^ in the Sho-saiko-to group, increased from 0.92 to 1.06 mg·h·L^−1^ in the Saiboku-to group, but did not change in the Saireito group [101].

#### 3.5.6. Coexistence of Inducers and Inhibitors

Clinicians may be confused about the net effect if a potent CYP3A inducer and a potent CYP3A inhibitor are combined in the same therapeutic regimen. Coadministration of St. John's wort (inducer) 300 mg t.i.d. and ritonavir (inhibitor) 300 mg twice daily for 14 days resulted in predominance of enzyme inhibition: AUC_(0–8 h)_ of intravenous midazolam increased to 180% of baseline value and AUC_(0–6 h)_ of oral midazolam increased to 412% of baseline value (*P* < 0.05 for each). At 2 days after discontinuation of the coadministered St. John's wort and ritonavir, oral midazolam AUC_(0–6 h)_ and intravenous midazolam AUC_(0–8 h)_ decreased to 6% and 33% of the level during combined administration, respectively [102]. It indicates that substantial dose adjustments may be required especially for oral CYP3A substrates when combined administration of St. John's wort and ritonavir is initiated or withdrawn. A comparative study showed that* Echinacea purpurea* 500 mg t.i.d for 28 days could significantly induce CYP3A activity but could not alter lopinavir-ritonavir exposure in healthy subjects. The most likely explanation for this phenomenon may be the fact that ritonavir, a potent intestinal and hepatic CYP3A inhibitor, masked the CYP3A-inducing effects of* Echinacea purpurea* on CYP3A substrate lopinavir.* Echinacea purpurea* is assumed to unlikely affect the pharmacokinetics of ritonavir-boosted protease inhibitors [103].

## 4. Conclusions

This review of HDIs from the perspectives of evidence based on randomized controlled trials and clinical risk management will enrich the knowledge of safety, efficacy, and economics-oriented therapeutics as well as research opportunities. Nevertheless, patients are complex and this complexity results from biological, medical (e.g., gender, age, genetics, polypharmacy, multimorbidities, and adherence), socioeconomic, and cultural factors (e.g., beliefs, values, and traditions). Clinical professionals should strengthen risk management on combination use of herbal medicines and conventional medicines such as increasing awareness of potential changes in therapeutic risk and benefits, inquiring patients about all currently used conventional medicines and herbal medicines and supplements, automatically detecting highly substantial significant HDI by computerized reminder system, selecting the alternatives, adjusting dose, reviewing appropriateness of the whole regimen, educating patients to monitor for drug-interaction symptoms, and paying attention to follow-up visit and consultation. The extent of an HDI may be associated with the factors (e.g., pharmacogenomics, dose of active ingredients in herbs, time course of interaction, characteristics of the object drugs such as administration routes and pharmacokinetic profiles, modification of herbal prescription compositions, and coexistence of inducers and inhibitors in the same therapeutic regimen), and these factors should be considered in the future RCTs investigating HDIs (if applicable).

## Figures and Tables

**Table 1:** Herb-drug interactions based on randomized controlled trials.

Significant pharmacokinetic HDIs with potential or actual clinical relevance	Significant pharmacodynamic HDIs with potential or actual clinical relevance	Neutral HDIs lack of potential clinical relevance
Baicalin + rosuvastatin [11]	Free and Easy Wanderer Plus + carbamazepine [46]	Liu Wei Di Huang Wan + substrates of CYP2C19, CYP2D6, or CYP3A4 (cocktail phenotyping study) [61]
St. John's wort + warfarin [13, 14]	Shakuyaku-kanzo-to + antipsychotic agents [47]	*Citrus aurantium*, *Echinacea purpurea*, milk thistle, saw palmetto extracts, *Ginkgo biloba*, or *Panax ginseng* + substrates of CYP1A2, CYP2D6, CYP2E1, or CYP3A4 (cocktail phenotyping study) [32, 33, 62]
St. John's wort + oral oxycodone [15]	Saffron aqueous extract + olanzapine [48]	Curcuminoid/piperine extract + substrates of CYP3A, CYP2C9, UGT, or SULT (cocktail phenotyping study) [63]
St. John's wort + oral S-ketamine [18]	Saffron + fluoxetine [49, 50]	St. John's wort + substrates of CYP3A4, CYP1A2, CYP2C9, or P-gp (cocktail phenotyping study) [64]
St. John's wort + cyclosporine [19]	*Ginkgo biloba* extract + haloperidol [51]	Woohwangcheongsimwon suspension + bupropion (CYP2B6 phenotyping study) [65]
St. John's wort + irinotecan [20]	Cranberry + warfarin [52]	*Ginkgo biloba* extracts + flurbiprofen (CYP2C9 phenotyping study) [66]
*Echinacea* + warfarin [23]	*Ginkgo biloba* extract + iodine-131 therapy [53]	Milk thistle and black cohosh supplementation + digoxin (P-gp phenotyping study) [67]
American ginseng + warfarin [24]	Radix/rhizoma notoginseng extract + aspirin [54]	Silibinin + nifedipine (CYP3A4 phenotyping study) [68]
*Ginkgo biloba* extracts + simvastatin [25]	Bergamot polyphenolic fraction + rosuvastatin [55]	Turmeric extract + nifedipine (CYP3A4 phenotyping study) [69]
*Eurycoma longifolia* water-based extract + propranolol [26]	Total ginsenosides + ulinastatin [56]	Grape seed extract + dextromethorphan (CYP2D6 phenotyping study) [70]
Yin Zhi Huang + omeprazole [27]	Fuzheng Yiliu decoction + chemotherapy [57]	Radix Astragali extract + fexofenadine (P-gp phenotyping study) [71]
Dehusked *Garcinia kola* seed + quinine [29]	St. John's wort + low-dose oral contraceptive [58]	Hawthorn preparation + digoxin (P-gp phenotyping study) [72]
Berberine + cyclosporine [30]	St. John's wort product + atorvastatin or simvastatin [59, 60]	Standardized goldenseal, kava-kava, milk thistle, or black cohosh supplement + digoxin (P-gp phenotyping study) [73, 74]
St. John's wort + substrates of CYP3A (phenotyping study) [31–33]		St. John's wort + repaglinide or boceprevir [75, 76]
St. John's wort + substrates of P-gp (phenotyping study) [34–36]		*Ginkgo biloba* extracts + ticlopidine, cilostazol, clopidogrel, voriconazole, or raltegravir [77–81]
St. John's wort + substrates of CYP2C19 (phenotyping study) [37, 38]		*Ginkgo* or ginger + warfarin [82]
Effect of *Ginkgo biloba* extracts on P-gp [39]		Aged garlic extract + warfarin [83]
*Rhodiola rosea* + substrates of CYP2C9 (phenotyping study) [40]		Garlic extract + ritonavir [84]
Goldenseal + substrates of CYP3A or CYP2D6 (phenotyping study) [41, 42]		*Panax ginseng* + warfarin [86]
Genistein + substrates of CYP3A or P-gp (phenotyping study) [44]		Milk thistle extract + indinavir [87]
Berberine + substrates of CYP2D6, CYP2C9, and CYP3A4 [45]		Lavender oil preparation (Silexan) + oral contraceptive containing ethinyl estradiol and levonorgestrel [88]
		Sho-saiko-to, Rikkunshito, or Saireito + ofloxacin [89]
		Tong Xin Luo, Nao Xin Tong, Guan Mai Ning, or Yin Xing Ye + simvastatin [90]
		Paeoniae Radix + valproic acid [91]

*Notes*. P-gp, P-glycoprotein; CYP, cytochrome P450; UGT, UDP-glucuronosyltransferase; SULT, sulfotransferase.
